# Study of the optimum fluoroscopic angle for the implant fixation test of a leadless cardiac pacemaker

**DOI:** 10.1007/s10554-022-02686-w

**Published:** 2022-09-07

**Authors:** Jiang-Hua Zhang, Cheng Chen, Qiang Xing, Xian-Hui Zhou, Yao-Dong Li, Ling Zhang, Yan-Mei Lu, Zu Kela Tu-Erhong, Xu Yang, Xing-Bin Liu, Bao-Peng Tang

**Affiliations:** 1 Department of Pacing and Electrophysiology, The First Affiliated Hospital of Xinjiang Medical University, No. 137 Liyushan South Road, Urumqi, 830054 Xinjiang Uygur Autonomous Region China; 2 Department of Cardiac Electrophysiology and Remodeling, The First Affiliated Hospital of Xinjiang Medical University, Urumqi, Xinjiang Uygur Autonomous Region China; 3 Department of Cardiology, West China Hospital of Sichuan University, 37 Guo Xue Xiang, Chengdu, 610041 Sichuan China

**Keywords:** Leadless pacemaker, Micra, Fluoroscopic angle, Fixation test, Fluoroscopy duration

## Abstract

The Micra TPS™ (Medtronic) is the first leadless pacemaker listed in China. The best fluoroscopic angle for the intraoperative fixation test is selected according to different implantation sites to reduce the fluoroscopy duration and radiation dose, and the test is based on the early safety and effectiveness of the device after implantation. A total of 110 patients who underwent Micra TPS™ implantation were selected. Eighty patients were in group A, and 30 patients were in group B. Under the guidance of the conclusions from group A, the fluoroscopy duration, radiation dose and number of fluoroscopic positions of the best fluoroscopic angle of the fixation test according to different positions of the implanted interventricular septum were compared. In 85.0% of the group A implants, these angles were based on the right interior oblique (RAO) angle, with 48.5% cranial (CRA) and 29.4% caudal (CAU) angles. The angle of the tilting head side of the RAO angle was prioritized in group B, and referring to the average angle data, the average fluoroscopy duration for finding the best angle of fixation test was 1.7 ± 0.6 vs. 3.2 ± 1.8 min (P < 0.001), the average radiation dose was 270.4 ± 56.3 vs. 338.1 ± 112.9 mGy (P = 0.002), and the average number of fluoroscopic positions was 2.2 ± 0.6 vs. 4.2 ± 2.1 (P < 0.001), which was significantly less than that in group A. This study found that there was regularity in the fluoroscopic angle for the fixation test during Micra TPS™ operation.

*Level of Evidence* Level 3, local nonrandom sample.

## Introduction

Traditional transvenous pacemakers have some complications related to implantation, electrodes and pulse generators, such as electrode dislocation, electrode rupture, venous thrombosis, tricuspid regurgitation, and infection after implantation. It is usually necessary to remove the whole pacing system completely [1, 2]. The Micra TPS™ (Medtronic) is the first leadless cardiac pacemaker listed in China. Previous clinical studies have confirmed its safety and effectiveness [3–5]. The most important step in the process of Micra TPS™ implantation is that the fixation test (pull and hold) should be used to select an appropriate fluoroscopic angle to fully expose the 4 flexible nitinol tines to ensure that at least 2 flexible nitinol tines are inserted into the myocardial tissue; therefore, during the operation, the operator must identify different fluoroscopic angles to clearly expose the 4 flexible nitinol tines, resulting in an excessive radiation dose and prolonged operation time. Currently, there is no research regarding how to choose different fluoroscopic angles according to different implantation sites for the fixation test of the Micra TPS™. On the basis of the early safety and effectiveness in 80 patients (group A) in the early stage at the center, this study systematically observed how to choose the best fluoroscopic angle according to different implantation sites for the fixation test and verified the angle in the process of implantation in 30 cases (group B) in the later stage to provide a reference for the specific intraoperative operation.

## Methods

### Study design and patient population

In this investigator-initiated observational study, we analyzed all of the implantation procedures associated with Micra TPS™ implantation. A total of 110 patients with a Micra TPS™ implanted in our hospital from December 18, 2019, to August 1, 2021, were included. The average age of 80 patients (group A) was 71.6 ± 12.6 years old, and 49 were men (61.3%). The average age of the 30 patients (group B) was 71.2 ± 10.3 years old, and 13 were men (43.3%) (Table 1). All patients met the indications for VVI pacemaker implantation. The main criteria were as follows: (1) patients with single-chamber ventricular pacemaker indications, such as atrial fibrillation with atrioventricular block; (2) patients who could not receive a pacemaker implanted via a traditional approach because of pacemaker infection or other factors; (3) patients with an indication for a dual-chamber pacemaker implant but with an expected pacing ratio less than 20%; and 4) patients who were willing to receive a leadless pacemaker. All patients signed an informed consent form before the operation. Cardiac ultrasound, chest X-ray and other related preoperative examinations were completed. The study was reviewed and approved by the local ethics committees and conducted according to the principles of the Declaration of Helsinki.

**Table 1:** Patient baseline characteristics

Patient characteristic	Total (n = 110)	A group (n = 80)	B group (n = 30)	P
Age (years)	71.5 ± 12.0	71.6 ± 12.6	71.2 ± 10.3	0.88
Man sex (n)	62 (56.4%)	49 (61.3%)	13 (43.3%)	0.09
Body mass index (kg/m^2^)	24.6 ± 3.9	24.6 ± 4.0	24.5 ± 3.6	0.94
PM indication				
Sick sinus syndrome (n)	53 (48.2%)	38 (47.5%)	15 (50.0%)	0.82
Third-degree AV block (n)	16 (14.5%)	13 (16.3%)	3 (10.0%)	0.60
Second-degree AV block (n)	18 (16.4%)	12 (15.0%)	6 (20.0%)	0.73
High AV block (n)	14 (12.7%)	9 (11.3%)	5 (16.7%)	0.66
Sick sinus syndrome and AV block (n)	9 (8.2%)	8 (10.0%)	1 (3.3%)	0.46
Patient comorbidities				
Atrial fibrillation	27 (24.5%)	23 (28.8%)	4 (13.3%)	0.09
Coronary artery disease (n)	46 (41.8%)	34 (42.8%)	12 (40.0%)	0.81
Arterial hypertension (n)	70 (63.6%)	53 (66.3%)	17 (56.7%)	0.35
Diabetes (n)	32 (29.1%)	26 (32.5%)	6 (20.0%)	0.20
Dyslipidemia (n)	8 (7.3%)	6 (7.5%)	2 (6.7%)	0.79
Valvular heart disease (n)	14 (12.7%)	8 (10.0%)	6 (20.0%)	0.28
Pulmonary hypertension (n)	25 (22.7%)	16 (20.0%)	9 (30.0%)	0.27
COPD	16 (14.5%)	13 (16.3%)	3(10.0%)	0.60
Chronic kidney disease (eGFR < 60 mL/min) (n)	3 (2.7%)	3 (3.8%)	0	0.68
Echocardiography data				
LVEDD (mm)	48.2 ± 4.3	47.9 ± 4.2	48.9 ± 4.5	0.31
LVEF (%)	61.5 ± 3.4	61.7 ± 3.1	60.9 ± 3.9	0.27
Oral anticoagulants (n)	20 (18.2%)	16 (20.0%)	4 (13.3%)	0.42
Oral antiplatelet drug (n)	42 (38.2%)	32 (40.0%)	10 (33.3%)	0.52
Pacing system infection (n)	7 (6.4%)	6 (7.5%)	1 (3.3%)	0.72

*AV* atrioventricular, *eGFR* estimated glomerular filtration rate, *LVEDD* left ventricular end-diastolic diameter, *LVEF* left ventricular ejection fraction, *PM* pacemaker, *COPD* chronic obstructive pulmonary disease

### Implantation procedure

**Fig. 1:**
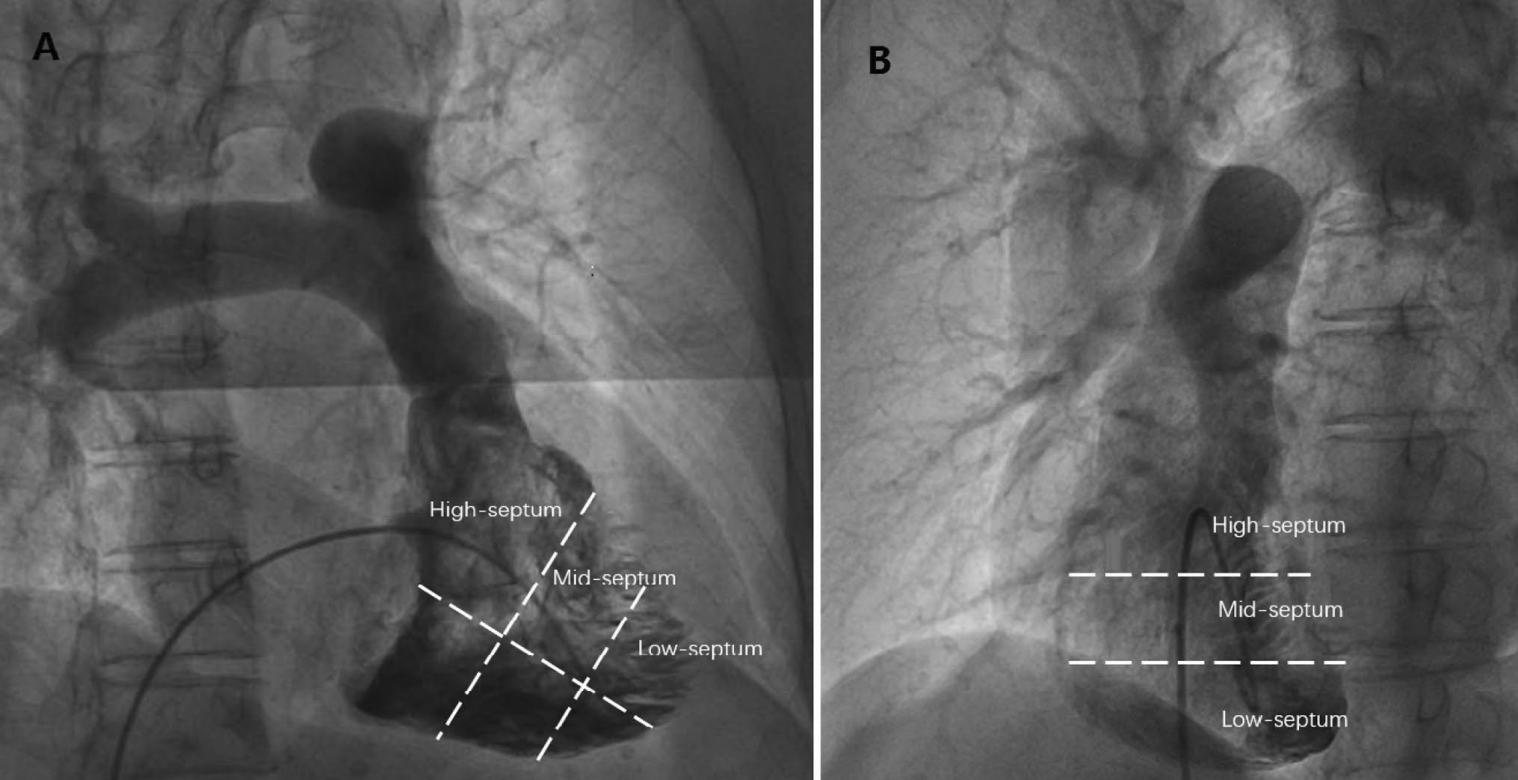
Right anterior oblique 30° right ventricular angiography (**A**) and left anterior oblique 45° right ventricular angiography (**B**)

There is a well-established procedure regarding the implantation of the Micra TPS™ [6]. We performed right ventriculography at 30° in the right anterior oblique (RAO) position and 45° ± 5° in the left anterior oblique position (LAO) during implantation to understand the right septal anatomy by standard fluoroscopy systems (UNIQ FD20C, Philips, Amsterdam, MA, the Netherlands). Along the RAO 30° coronal plane (Fig. 1A) and the LAO 45° sagittal plane (Fig. 1B), the lower part of the pulmonary valve is the upper limit of the interventricular septum, and the long axis of the apical interventricular septum is the lower limit. The interventricular septum was divided into high, median and low parts. To determine the location of the specific Micra TPS™ in the septum after implantation, in group A, the fixation test was performed by pulling on the tether until the heart beat was felt and then video recorded at 15 frames in a magnified frame for 2–3 s. The video was then reviewed frame by frame to identify tines that opened 10°–30° or more. If fewer than two tines were observed to open, then the fixation test could be repeated with more tension and/or at another fluoroscopic angle. We considered both LAO and RAO angulation, as well as cranial caudal angulation, since the Micra can lie in many orientations. Movement of two of the four tines was all that was required to determine adequate fixation; otherwise, the Micra was repositioned. Group B included 30 patients in whom the order of fluoroscopic angle selection was determined. First, we considered an RAO of 30°, magnification and appropriate increases in radiation dose to observe whether the four tines were clearly visible and more than two tines appeared to show open and closed movement; otherwise, we performed fine adjustment on the basis of an RAO angle of 20°–40°, successively adjusted within the range of a CRA angle of 10°–20° and a CAU of 10°–20°. If we still could not achieve the ideal observation effect, we finally considered an LAO angle of 10°–30° and combined the above CRA and CAU angles for perspective.

### Definition of the best fluoroscopic angle in the fixation test

After the Micra TPS™ is released from the release cup, it can fully expose the four nitinol tines under fluoroscopy and allow for full observation of the movement of the four nitinol tines during the fixation test.

### Definition of finding the best fluoroscopic angle radiation dose

The fixation test process was performed to clearly observe the four nitinol tines and fluoroscopic start, after a series of fluoroscopic angle adjustments and exposure confirmations, until the total amount of radiation was administered during the observation of at least 2 nitinol tines with the draft exhibiting an opening and closing motion.

### Filming protocol

The exposure mode that we chose mainly included a tube voltage of 60–120 kV, a tube current of 1–866 mA, and an imaging frame rate of 15 frames/s.

### Description and parameter comparison of the best fluoroscopic angle

To facilitate recording and description, we set the Cartesian plane coordinate system with the human heart as the origin and described the LAO and RAO angle positions as the positive and negative axes of the X-transverse axis, respectively. The CRA and CAU positions were the Y-longitudinal axis positive half axis and negative half axis, respectively. The best fluoroscopic angle in 80 patients (group A) when four Micra nitinol tines were fully exposed during the operation was recorded, and the mean, standard deviation and statistical mode were calculated. In the later stage, the implantation of 30 patients (group B) was based on the summary and application of the angle rule of group A and compared to determine whether there were significant differences in the fluoroscopy duration, radiation dose and number of fluoroscopic positions between the two groups during the fixation test.

### Statistical analysis

All data were analyzed using SPSS (version 25.0) software, and the categorical variables are expressed as numbers and corresponding percentages. If the numerical data were in accordance with a normal distribution, they were expressed as the ${\overline{\text{x}}}$ ± s, mode, etc., and if they were skewed, they were expressed as the median, quartile spacing, etc. The quantitative data were compared by the t test. Using the bilateral test, the difference was considered statistically significant at P < 0.05.

## Results

### Study patients

The average age of the 110 patients was 71.5 ± 12.0 years old, and 62 men were included in the study (56.4%). Multiple comorbidities were combined, including hypertension (63.6%), diabetes (29.1%), coronary artery disease (41.8%), and COPD (14.5%). A total of 6.4% of patients had a previous conventional pacemaker implant. The most common indication for pacing was sick sinus node syndrome (48.2%). There was no significant difference in baseline data between group A and group B (P > 0.05) (Table 1).

### Implantation condition

A Micra TPS™ was successfully implanted in all 110 patients as follows: in the low interventricular septum in 14 patients (12.7%), median interventricular septum in 60 patients (54.5%), and high interventricular septum in 36 patients (32.7%). A total of 81.8% (90/110) of the patients were successfully implanted with one deployment, and the average number of deployments in the 110 patients was 1.3 ± 0.8. After choosing the best fluoroscopic angle for the fixation test, all of the patients met the electrical test requirements. The mean pacing threshold was 0.5 ± 0.2 V at 0.24 ms, the mean R-wave amplitude was 10.5 ± 4.0 mV, and the mean impedance was 945.6 ± 253.7 Ω.

### The best fluoroscopic angle

In group A, when the low interventricular septum was the implant site, 91.7% of the Micra TPS™ implants were located horizontally and longitudinally, in the RAO and CRA mainly. When the median interventricular septum was the implant site, 41.0% of the implants had a single angle, with an RAO angle of 30° in 50.0% of these implants accounting for the majority; 59.0% of the Micra TPS™ implants had both horizontal and vertical angles. Overall, RAO, CAU and CRA angles dominated. When the high interventricular septum was the implant site, 93.1% of the implants had both horizontal and longitudinal angles, mainly RAO and CRA (Table 2).

**Table 2:** Micra TPS™ fluoroscopic angle during low, median and high interventricular septal fixation tests

	Low (n = 12)			Median (n = 39)			High (n = 29)		
	Ratio	Mean	Multiplicity	Ratio	Mean	Multiplicity	Ratio	Mean	Multiplicity
RAO	10/12	31 ± 13	15(2/10)	33/39	28 ± 7	30(14/33)	25/29	27 ± 9	30(6/25)
			30(2/10)						20(4/25)
LAO	1/12	–	–	4/39	28 ± 10	20(2/4)	3/29	13 ± 11	20(2/3)
CRA	7/12	19 ± 11	10(3/7)	12/39	17 ± 6	20(4/12)	20/29	15 ± 8	15 (6/20)
									10 (4/20)
CAU	5/12	17 ± 13	10(2/5)	13/39	21 ± 7	20(5/13)	7/29	16 ± 8	–
AP	–	–	–	–	–	–	1/29	–	–

*RAO* right anterior oblique, *LAO* left anterior oblique, *CRA* cranial, *CAU* caudal, *AP* anterior posterior

**Fig. 2:**
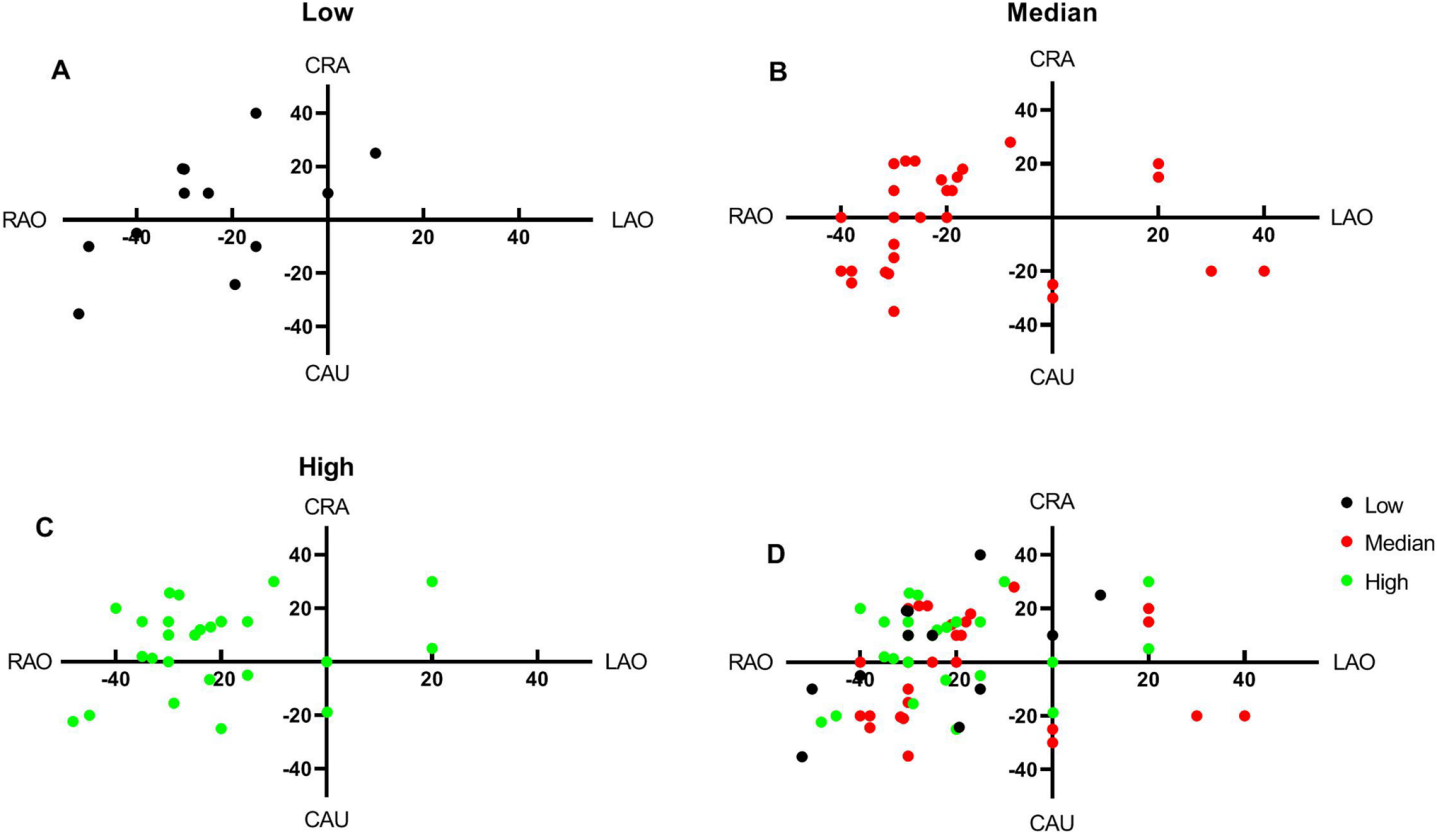
Fluoroscopic angles of the Micra TPS™ implanted in the low (**A**), median (**B**), and high (**C**) interventricular septal fixation tests and the total (**D**)

We further plotted the coordinate scatter diagram. When the Micra TPS™ was implanted in the low interventricular septum (Fig. 2A), 83.3% of the implants had a fluoroscopic angle based on the RAO position, of which 50.0% of the implants had CRA angles, and 50.0% had CAU angles; when the Micra TPS™ was implanted in the median interventricular septum (Fig. 2B), 84.7% of the implants had a fluoroscopic angle based on the RAO position, of which 30.3% had CRA angles, and 27.3% had CAU angles. When the Micra TPS™ was implanted in the high interventricular septum (Fig. 2C), 86.2% of the implants had a fluoroscopic angle based on the RAO position, of which 72.0% had CRA angles, and 24.0% had CAU angles. Overall, the fluoroscopic angles of the vast majority of the implants (85.0% (68/80)) were located in the second and third quadrants of the coordinate system (Fig. 2D) and were based on the RAO position; specifically, 48.5% (33/68) of these implants had CRA angles, and 29.4% (20/68) had CAU angles.

In group B, 30 patients had the following characteristics: low interventricular septum in 2 patients, median interventricular septum in 21 patients, and high interventricular septum in 7 patients.

All of the implants used the RAO angle, and 16.7% of the implants had a single angle, with an RAO angle of 30°. Of the Micra TPS™ implants, 83.3% had both horizontal and vertical angles, where the RAO/CRA ratio was 17/25, and the RAO/CAU ratio was 8/25. In general, the X-horizontal axis of the implants was represented by an average RAO angle of 28 ± 7°, while an average CRA angle of 19 ± 5° and a CAU angle of 19 ± 4° were observed for the Y-vertical axis of the implants. The difference was not statistically significant with group A (P > 0.05).

### Intraoperative fluoroscopy duration parameters for finding the best fluoroscopic angle

**Fig. 3:**
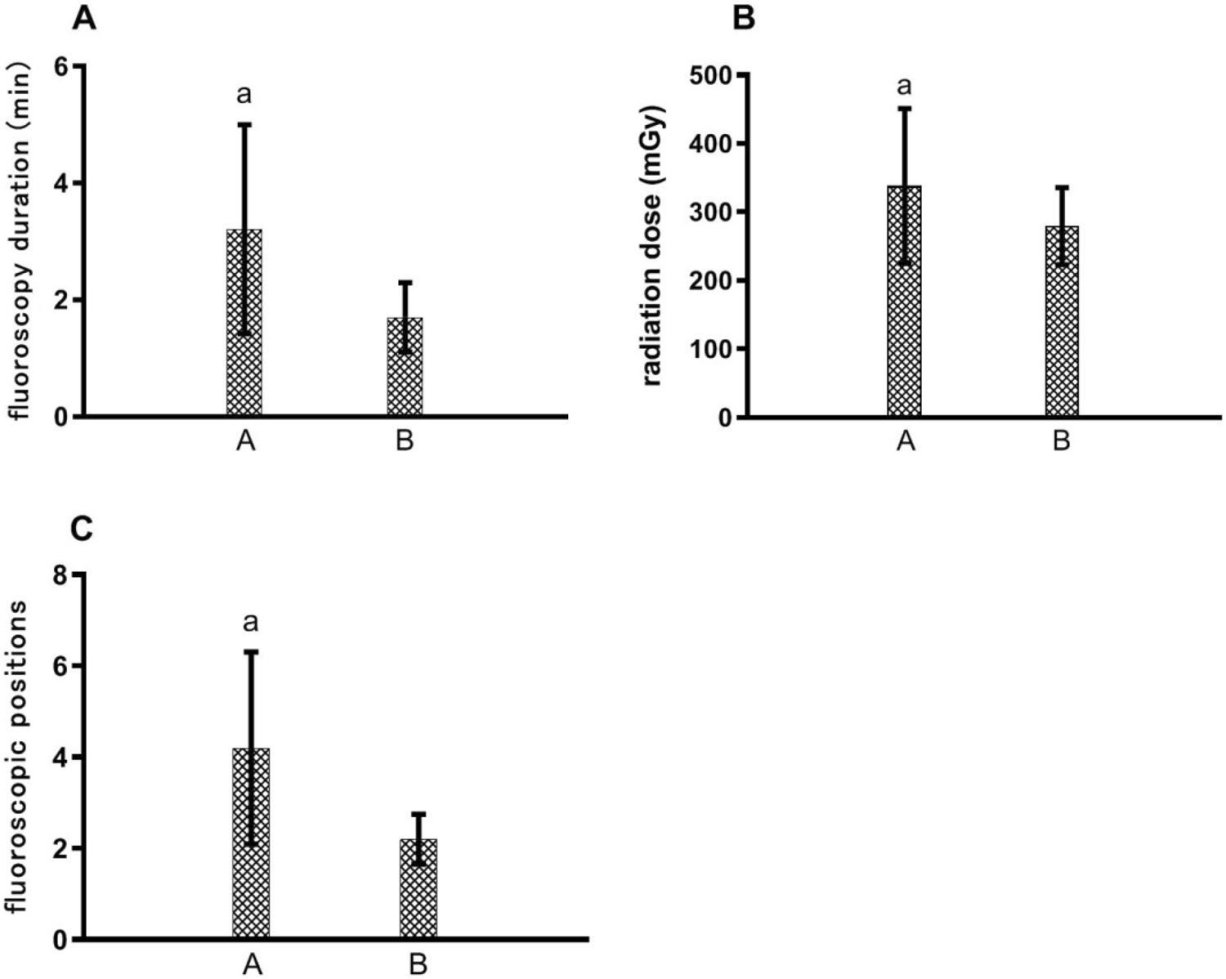
Fluoroscopy duration (**A**), radiation dose (**B**), and number of fluoroscopic positions (**C**) in the process of finding the best fluoroscopic angle. ^a^Significant difference compared with other groups; A = 80 cases in the early stage and B = 30 cases in the late stage

The mean operation time, fluoroscopy duration and radiation dose for Micra TPS™ implantation in the 110 patients were 41.2 ± 15.2 min, 8.8 ± 2.6 min and 627.8 ± 167.5 mGy, respectively. The X-ray exposure intensity, magnification and radiation dose were higher than those for ordinary fluoroscopy and film. In group A, the average fluoroscopy duration for finding the best fluoroscopic angle and completing the fixation test was 3.2 ± 1.8 min, the average radiation dose was 338.1 ± 112.9 mGy, and the average number of fluoroscopic positions was 4.2 ± 2.1. The data from group B were as follows: compared with group A, the average fluoroscopy duration was 1.7 ± 0.6 min (P < 0.001), the average radiation dose was 270.4 ± 56.3 mGy (P = 0.002), and the average number of fluoroscopic positions was 2.2 ± 0.6 (P < 0.001). There were significant differences in fluoroscopy duration (Fig. 3A), radiation dose (Fig. 3B) and number of fluoroscopic positions (Fig. 3C) between the two groups. With increased operator familiarity with the best fluoroscopic angle, the fluoroscopy duration, radiation dose and number of fluoroscopic positions were significantly decreased.

## Discussion

The fluoroscopic angles of 80 early patients with Micra TPS™ intraoperative fixation tests implanted in our center were summarized. When low interventricular septal implantation was selected, an RAO angle of 31° ± 13° and a CRA angle of 19° ± 11° were selected. According to the scatter plot of the best fluoroscopic angle, most of the best fluoroscopic angles were located in the second and third quadrants of the coordinate system; that is, the RAO angle was mostly located at 20°–40°, and the CRA-CAU position was located at 10°–20°. When median interventricular septal implantation was chosen, an RAO angle of 28° ± 7° and a CAU angle of 21° ± 7° or a CRA angle of 16° ± 7° were the first choice; similar to low septal implantation, most of the best fluoroscopic angles for median septal implantation were located in the second and third quadrants of the coordinate system. When high interventricular septal implantation was used, an RAO angle of 27° ± 9° and a CRA angle of 15° ± 8° were the first choices. According to the scatter diagram of the best fluoroscopic angles, although most of the best fluoroscopic angles for high septal implantation were located in the second and third quadrants of the coordinate system, the number of cases in the second quadrant was significantly larger than that in the third quadrant (18/29 vs. 6/29); that is, the four nitinol tines were more easily observed with RAO and CRA angles. Generally, whether in the low, median or high position, the best fluoroscopic angle was 85% of the RAO position plus the CRA and CAU positions, and the RAO position was generally approximately 30°. According to the optimal fluoroscopic angle of the fixation test summarized in the previous stage, we implanted 30 Micra TPS™ pacemakers in the later stage. The results showed that, similar to each patient in group A, most of the best fluoroscopic angles were located in the second and third quadrants with the RAO angle plus the head or foot position. An RAO angle of 27° ± 7° and a CRA angle of 19° ± 5° were the first choices. The difference was not statistically significant with group A (P > 0.05). Finding the best fluoroscopic angle became easier compared to group A; the fluoroscopy duration was reduced by approximately 2–3 min, and the radiation dose was reduced by approximately 100 mGy. The number of fluoroscopic positions needed to find the best fluoroscopic angle was reduced to 2–3 positions.

Compared with that for a traditional single-chamber pacemaker, the operation time for Micra TPS™ implantation is significantly shorter, but the radiation dose and fluoroscopy duration are significantly higher and longer, respectively [3, 4]. The Micra TPS™ was implanted into the right ventricular septum through the femoral vein using a 23-French outer sheath and was fixed by four flexible nitinol tines, which were firmly combined with the myocardial trabeculae of the right ventricle. The standard for Micra TPS™ to be reliably fixed is the fluoroscopic fixation test to ensure that more than 2 nitinol tines move together with the traction and that more than 2 nitinol tines are inserted into the myocardial tissue. This procedure requires an appropriate fluoroscopic angle to fully expose the four nitinol tines. The operator must choose different fluoroscopic angles repeatedly during the operation; ultimately, determining the number of myocardial nitinol tines with the best observation angle is an important reason for the prolonged fluoroscopy duration and high radiation dose.

Anatomically, the high interventricular septum is located in the area of the right ventricular outflow tract from the pulmonary valve to the supraventricular ridge; the median septum is located at the papillary muscle level from the supraventricular ridge to the right ventricular inflow tract; and the low septum is located at the level below the anterior papillary muscle [7]. However, the specific anatomical criteria cannot be distinguished from right ventriculography, so the right ventricular septum is equally divided into high, median and low parts at two relatively fixed angles. In terms of surgical safety and QRS duration after pacing, the low septum is not the best choice for Micra TPS™ implantation [8, 9]. In Micra TPS™ implantation in our center, the median and high septa constitute the first choice for the implant site, and the low septum is chosen when the electrical parameters are not superior. Therefore, in this study, the proportion of Micra TPS™ implants in the median and high septum positions was the largest. From the scatter view of the best fluoroscopic angle between the median and high positions, it can be seen that the high position is more likely to include the fluoroscopic CRA angle with the RAO angle.

**Fig. 4:**
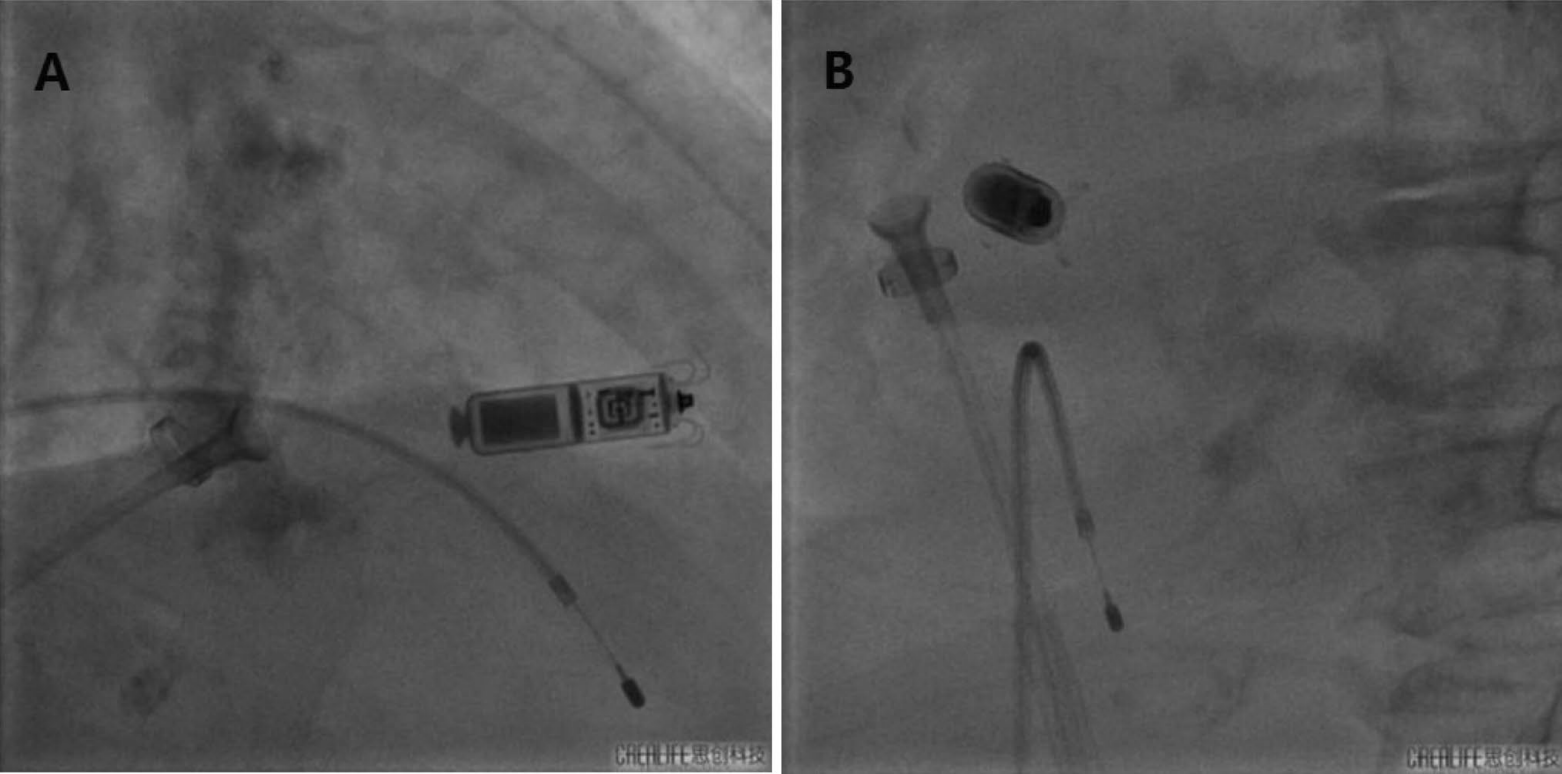
RAO position exposes the long axis of the four nitinol tines (**A**), LAO position exposes the short axis of the four nitinol tines (**B**)

The best fluoroscopic angle is mainly in the RAO position, which is closely related to the relative spatial position and relative angle between the sheath and the interventricular septum of the Micra TPS™ during the operation. Due to the acute angle between the Micra TPS™ implant and the spacer surface, the right front oblique position is the long axis surface of the Micra TPS™ fuselage and the nitinol tines, maximizing the opening and closing motions of the four nitinol tines during the fixation test (Fig. 4A). While the left front oblique position is the short axis surface of the Micra TPS™ fuselage and the nitinol tines, it can also expose the four nitinol tines, although it shortens the fixed nitinol tines. Visually, it was not easy to observe the opening and closing motions of the fixed nitinol tines during the fixation test (Fig. 4B).

Because the space position of the four nitinol tines is not fixed when the Micra TPS™ is released, the RAO position must in theory be adjusted cranially or caudally to clearly see the four nitinol tines. On this basis, fine tuning within the average angle range will enable the operator to find the best fluoroscopic angle more quickly to effectively reduce the radiation dose and fluoroscopy duration and further optimize the operation. The experience of our center is to find the best CRA or CAU fluoroscopic angle on the basis of the RAO angle.

## Study limitations

This sample was composed of Micra TPS™ implantation patients from a single center, the sample size was small, and there might not have been sufficient data to show the law of angles accurately. Many of the patients had a cardiac transposition, so each patient had a special best observation angle; sometimes, there can be more than two best fluoroscopic angles. In addition, in some patients, the movement of a sufficient number of fixed nitinol tines in one position could not be observed, and fixation tests with multiple fluoroscopic angles were needed to confirm the number of active fixed nitinol tines.

## Conclusion

During the Micra TPS™ operation, the fluoroscopic angle of the fixation test had a certain regularity. With operator familiarity regarding finding the best fluoroscopic angle regularity, the fluoroscopy duration, radiation dose and number of fluoroscopic positions gradually decreased and tended to be stable.

## Data Availability

The data that support the findings of this study are available from the corresponding author upon reasonable request.

## Abbreviations

AV: Atrioventricular
eGFR: Estimated glomerular filtration rate
LVEDD: Left ventricular end-diastolic diameter
LVEF: Left ventricular ejection fraction
PM: Pacemaker
COPD: Chronic obstructive pulmonary disease
RAO: Right anterior oblique
LAO: Left anterior oblique
CRA: Cranial
CAU: Caudal
AP: Anterior posterior
